# Treatment with the SQ tree sublingual immunotherapy tablet is safe and well tolerated in real‐life

**DOI:** 10.1002/clt2.12373

**Published:** 2024-07-02

**Authors:** Oliver Pfaar, Hendrik Wolf, Rainer Reiber, André Knulst, Kirsten Sidenius, Mika J. Mäkelä, Sverre Steinsvåg, Christer Janson, Leonard van der Zwan, Elena Uss, Peter Arvidsson, Kathrin Borchert, Helena Himmelhaus, Eike Wüstenberg

**Affiliations:** ^1^ Department of Otorhinolaryngology, Head and Neck Surgery, Section of Rhinology and Allergy University Hospital Marburg Philipps‐Universität Marburg Marburg Germany; ^2^ Medical Department/Clinical Development ALK‐Abelló Arzneimittel GmbH Hamburg Germany; ^3^ Facharztpraxis für HNO/Allergologie Schorndorf Germany; ^4^ Department of Dermatology/Allergology University Medical Center Utrecht Utrecht University Utrecht The Netherlands; ^5^ Allergiklinikken i Bagsværd Bagsværd Denmark; ^6^ Aleris Hospitaler København Søborg Denmark; ^7^ Skin and Allergy Hospital HUS Helsinki University Hospital Helsinki Finland; ^8^ Sørlandet Sykehus Kristiansand Norway; ^9^ Department of Medical Sciences: Respiratory, Allergy and Sleep Research Uppsala University Uppsala Sweden; ^10^ ALK‐Abelló Benelux Almere The Netherlands; ^11^ ALK Nordic A/S Danmark filial Kungsbacka Sweden; ^12^ Xcenda GmbH part of Cencora Hannover Germany; ^13^ Department of Otorhinolaryngology Head and Neck Surgery Faculty of Medicine (and University Hospital) Carl Gustav Carus Technische Universität Dresden Dresden Germany

**Keywords:** allergen immunotherapy, post‐authorisation safety study, real‐life, SQ tree‐SLIT‐tablet, tolerability

## Abstract

**Background:**

The SQ tree sublingual immunotherapy (SLIT)‐tablet is authorised for treatment of allergic rhinoconjunctivitis with or without asthma in trees of the birch homologous group in 21 European countries. The primary objective of this study was to explore the safety in real‐life.

**Methods:**

In a prospective, non‐interventional post‐authorisation safety study (EUPAS31470), adverse events (AEs) and adverse drug reactions (ADRs) at first administration and follow‐up visits, symptoms, medication use, and pollen food syndrome were recorded by physicians in 6 European countries during the first 4–6 months of treatment.

**Results:**

ADRs with the SQ tree SLIT‐tablet were reported in 57.7% of 1069 total patients (median age 36.0 years, 53.7% female) during the entire observation period (severity, mild‐to‐moderate: 70.1%, severe: 4.7%, serious: 0.7%) and in 45.9% after first administration. ADRs were not increased with pollen exposure at first administration. With coadministration of the SQ tree and grass SLIT‐tablet AEs were reported in 73.8% of patients and in 52.8% with the SQ tree SLIT‐tablet alone. Nasal and eye symptoms improved in 86.9% and 80.9% of patients and use of symptomatic medication in 76.0%. PFS with symptoms was reported in 43.0% of patients at baseline and in 4.3% at the individual last visit.

**Conclusions:**

The results of this non‐interventional safety study with the SQ tree SLIT‐tablet confirm the safety profile from placebo‐controlled clinical trials and support effectiveness in real‐life according to the published efficacy data. Safety was not impaired by pollen exposure at first administration or co‐administration with other SLIT‐tablets.

## 1 INTRODUCTION

Pollen from birch and other birch‐related trees is one of the main allergen sources causing allergic rhinoconjunctivitis (ARC) in northern and central Europe, and in certain areas of North America.^1^, ^2^, ^3^

Birch‐related pollen allergy is also present in southern Europe and the Mediterranean countries, but the frequency of sensitisation is much lower.^4^, ^5^

Due to cross‐reactivity between allergens from birch, alder, hazel, hornbeam, oak and beech and certain foods, with apple and tree nuts as most frequent triggers,^6^ many tree allergic patients also develop oral allergy symptoms denoted as pollen food syndrome (PFS).

The SQ tree sublingual immunotherapy (SLIT)‐tablet has been developed for allergen immunotherapy (AIT) in patients with ARC to tree pollen. AIT is the only treatment with the potential for modifying the allergic disease.^7^, ^8^

The SQ tree SLIT‐tablet has been authorised for treatment of adults with ARC to allergens from trees of the birch homologous group in 21 European countries according to a clinical development program comprising four randomised, double‐blind, placebo‐controlled (RDBPC) clinical trials in phase I to III.

After a phase I‐trial (TT‐01),^9^ 12 SQ‐Bet was identified as the optimal dose for further development in two RDBPC‐trials in phase II, a field trial with 637 subjects in Europe (TT‐02),^10^ and an environmental exposure chamber trial in 219 subjects (TT‐03).^11^

The pivotal, phase III RDBPC‐trial (TT‐04) demonstrated efficacy and safety of the SQ tree SLIT‐tablet including 634 subjects (aged 12–65 years) with moderate‐to‐severe ARC despite use of symptom‐relieving medication^12^; a post hoc analysis demonstrated efficacy also during the oak pollen season.^13^

In general, treatment with the SQ tree SLIT‐tablet was well tolerated. The most frequently reported treatment‐related adverse events (AEs) were mild‐to‐moderate oral pruritus and throat irritation as local reactions related to the sublingual administration.^14^

In clinical trials, patients were solely treated with the tree SLIT‐tablet. In real‐life, since many patients are poly‐allergic to tree and grass pollen, co‐administration of tree‐ and grass‐AIT is common.

To investigate whether the safety and tolerability of the SQ tree SLIT‐tablet established by controlled clinical trials is similar in routine clinical practice, we conducted a voluntary, non‐interventional, post‐authorisation safety study (NIS‐PASS) in 6 northern and central European countries, including safety data on co‐administration with other SLIT‐tablets in real‐life.^15^

## 2 MATERIALS AND METHODS

### 2.1 Study design and treatment

This prospective, open‐label, observational NIS‐PASS was conducted by physicians across Germany, Denmark, Finland, The Netherlands, Norway, and Sweden. The study has been registered in the European Union electronic Register of Post‐authorisation Studies (EUPAS31470).

Data on treatment of patients with ARC with or without allergic asthma (AA) to tree pollen (birch, alder, hazel) were recorded using an electronic case report form (eCRF). Treatment was initiated with the SQ tree SLIT‐tablet (ITULAZAX^®^) in the clinic and continued by daily self‐administration of the patients at home. Physicians were asked to include 1 to 20 patients dependent on the patient's willingness to participate. To record representative data, patients were included by sites distributed across the 6 countries involved. To reduce a potential selection bias, physicians were instructed to include patients in a consecutive order according to patients' consent on participation. Data of up to four visits were recorded according to the physicians' routine practice, with a scheduled interval of 1–3 months.

### 2.2 Endpoints

Primary endpoint was the sum of local adverse drug reactions (ADRs) including pruritus, irritation, swelling or oedema at lips, mouth, palate, pharynx, and larynx. Secondary safety endpoints were the number of ADRs, non‐local, systemic (potential anaphylactic reactions), serious ADRs, and the sum of local ADRs in patients with a history of PFS. Effectiveness was evaluated as change in symptoms, use of symptomatic medication, and change in PFS.

### 2.3 Ethics and data protection

The study was approved by national authorities according to the laws of the participating countries and reviewed by responsible ethics committees (see Supporting Information S1).

The patients' written informed consent for collection, processing and use of their data was obtained. The physician's decision to prescribe the SQ tree SLIT‐tablet was taken independently from the inclusion of the patient in the study. Direct identification of the patients was restricted to the sites that participated in the study.

### 2.4 Patients

According to the summary of product characteristics, adult patients (aged 18–65 years) with moderate‐to‐severe allergic rhinitis and/or conjunctivitis induced by pollen from trees of the birch‐homologous group and diagnosed by clinical history and a positive test for sensitisation to tree pollen allergens (skin prick test and/or specific immunoglobulin E (IgE)) were included.^16^ Contraindications to treatment with the SQ tree SLIT‐tablet are: hypersensitivity to any of the excipients, forced expiratory volume in one second (FEV_1_) < 70% of predicted value (after adequate pharmacological treatment) at initiation of treatment, severe asthma exacerbation and uncontrolled asthma in the last 3 months before start of treatment, active systemic autoimmune diseases (not responding to treatment), immune defects, immunodeficiencies, immunosuppression or malignant neoplastic diseases with current disease relevance, and acute severe oral inflammation or oral wounds.^16^

### 2.5 Assessments

#### 2.5.1 AEs and ADRs

AEs were specified by the physician by severity (mild/moderate/severe), causality (possible/unlikely), change of treatment (no change/temporary interruption/discontinuation/dose reduced), treatment by medication, outcome (recovered/recovered with sequelae/not recovered/fatal/unknown) and seriousness (yes/no). The severity of an AE was assessed by the investigator using the following definitions: mild (no or transient symptoms, no interference with the patient's daily activities); moderate (marked symptoms, moderate interference with the patient's daily activities); severe (considerable interference with the patient's daily activities, unacceptable). A serious AE was defined as any medical occurrence or effect that was life‐threatening, required hospitalisation or prolongation of hospitalisation, resulted in persistent or significant disability or incapacity, resulted in death, was a congenital abnormality or birth defect, or any other event judged medically important. AEs with possible relation to treatment with the SQ tree SLIT‐tablet were specified as ADRs.

#### 2.5.2 Study visit 1

At study visit 1, data on demographics, allergy history, including age at first appearance of symptoms, clinical manifestations of the allergy (rhinitis/conjunctivitis/asthma/atopic dermatitis), other allergies, diagnostics performed and any current or previous treatment by AIT were recorded, and administration of any additional SLIT‐treatments (liquid drops or tablets) or other medications due to concomitant diseases. The severity of symptoms at nose, eyes, bronchi, and skin (no/mild/moderate/severe) and patients' use of anti‐allergic pharmacotherapy were assessed for the previous 12 months as baseline data. Accordingly, data on PFS were recorded (no PFS/PFS, no symptoms/PFS, symptoms within the last 12 months), specific symptoms of PFS, foods triggering PFS, and severity (mild/moderate/severe). The severity of symptoms (mild, moderate, severe) recorded in the eCRF followed the same definitions as the assessment of the severity of AEs.

First administration of the SQ tree SLIT‐tablet in the clinic was recorded with time and date, any symptom‐relieving premedication, and AEs during the 30‐min surveillance period.

#### 2.5.3 Follow‐up visits

Patients were scheduled to return for the follow‐up visits (visit 2, 3, 4) after 1–3 months according to the initial prescription of 30 or 90 tablets to renew their prescription following the routine procedure of the physician for a scheduled total observation period of 4–6 months. At follow‐up visits, AEs that occurred since the last visit, follow‐up information on previous AEs, and changes in the medical treatment of concomitant diseases were recorded as well as adherence to treatment with the SQ tree SLIT‐tablet (tablet taken on average since last visit: 7, 6, 5, 4, or ≤3 times per week and reasons, if not taken every day). The severity of symptoms at the nose, eyes, bronchi and skin, use of anti‐allergic pharmacotherapy and data on PFS were assessed at all visits. At the final visit, continuation or discontinuation with the date of last administration of the SQ tree SLIT‐tablet was recorded and the reasons for discontinuation.

### 2.6 Statistics

Data were summarised by descriptive statistics. No formal sample size calculation and statistical tests were performed. The sample size of approximately 1000 patients followed empirical considerations and was based on a need to detect a sufficient number of AEs to evaluate the safety profile of the SQ tree SLIT‐tablet in the real‐life setting.

All patients with at least one administration of the SQ tree SLIT‐tablet who fulfilled the eligibility criteria and with eCRF entries verified by the treating physician comprised the full analysis set (safety set). Demographics and other baseline characteristics were displayed with summary statistics (number of patients, minimum, maximum, mean, median, 25% and 75% percentiles) and frequency tables for categorical variables. AEs as well as breakdown of AEs and treatment‐related AEs (possibly related) according to seriousness, severity and causality were summarised for AEs during administration (visits 1 to 4) as ADRs.

AEs and ADRs were stratified into five subgroups according to the clinical manifestations of allergy: ARC; ARC and AA (ARC + AA); ARC and PFS (ARC + PFS); ARC, AA and PFS (ARC + AA + PFS); ARC and AD with or without AA and with or without PFS (ARC + AD (± AA ± PFS)).

The following periods were estimated as tree pollen seasons: February to June 2021 and January to June 2022 (Germany, Denmark, The Netherlands) and March to July 2021 and February to July 2022 (Finland, Norway, Sweden). Effectiveness data (symptoms at nose, eyes, bronchi, skin, and use of symptomatic medication) were analysed versus baseline in all patients with available data for their individual last visit and in patients during their individual last visit within the period of the estimated tree pollen season.

AEs and ADRs were coded according to the Medical Dictionary for Regulatory Activities (MedDRA, version 22.0 or higher) as System Organ Classes (SOCs) and Preferred Terms (PTs), displaying the number of patients, the percentage of total patients experiencing AEs and the number of events (e). Missing data were not replaced.

## 3 RESULTS

### 3.1 Demographic and baseline data

Ten to 20 patients per site (except for 2 sites with 40 patients in The Netherlands) were included by 112 sites in 6 countries (The Netherlands: 46 sites, Germany: 35, Denmark: 11, Norway: 11, Sweden: 7, Finland: 2). The flow of patients through the study is available in Figure S1.

Relevant previous and concomitant diseases were reported in 221 (20.7%) patients and in 211 (19.7%) as ‘ongoing’. Most frequent MedDRA PTs of concomitant diseases were asthma (7.1% of patients), hypertension (3.6%) and hypothyroidism (1.7%), depression, dermatitis atopic, eczema (1%, each), others (<1%). Relevant previous and concomitant medication was reported in 296 (27.7%) patients and in 286 (26.8%) as ‘ongoing’. Most frequent drugs classified according to WHO Drug Anatomical Therapeutic Chemical codes were R06: antihistamines for systemic use (12.3% of patients), R03: drugs for obstructive airway diseases (9.3%), R01: nasal preparations (8.7%), S01: ophthalmologicals (4.3%), D07: corticosteroids, dermatological preparations (3.3%), C09: agents acting on the renin‐angiotensin system (3.0%), others (<3.0%).

Demographic and baseline data (clinical manifestations of tree pollen allergy, symptoms and medication use during the previous 12 months), and data on allergy history of patients, for total patients and the 5 stratified subgroups (ARC, ARC + AA, ARC + PFS, ARC + AA + PFS, ARC + AD (± AA ± PFS)) are displayed in Table 1. At initiation of treatment with the SQ tree SLIT‐tablet, 119 patients were treated by another SLIT‐tablet with different allergens. The majority, 61 (51.3%) patients administered one tablet in the morning and the other in the evening, 25 (21.0%) patients with an interval of 30 min, 23 (19.3%) patients at the same time, and 10 (8.4%) with other intervals. Moderate‐to‐severe baseline nasal symptoms were reported in 95.5% of total patients, eye symptoms in 77.9%, bronchial symptoms in 25.6% and skin symptoms in 8.8%. Oral antihistamines and nasal corticosteroids were the most frequently used symptomatic medications, and, additionally, inhaled corticosteroids (ICS), short‐acting ß_2_‐agonists (SABA) and long‐acting ß_2_‐agonists (LABA) in the ARC + AA subgroup (Table 1). Median duration of treatment with the SQ tree SLIT‐tablet during the entire observation period of the study was 5.4 months (mean 156.6 (±66.0) days).

**TABLE 1:** Patient characteristics at baseline.

	All patients (*n* = 1069)	ARC (*n* = 332)	ARC + AA (*n* = 135)	ARC + PFS (*n* = 249)	ARC + AA + PFS (*n* = 200)	ARC + AD (± AA ± PFS) (*n* = 153)
Median age, y	36.0	35.0	41.0	36.0	41.0	33.0
Range, y	18.0–65.0	18.0–65.0	19.0–65.0	18.0–65.0	18.0–64.0	18.0–65.0
Sex, *n* (%)						
Male	495 (46.3)	177 (53.3)	66 (48.9)	120 (48.2)	91 (45.5)	41 (26.8)
Female	574 (53.7)	155 (46.7)	69 (51.1)	129 (51.8)	109 (54.5)	112 (73.2)
BMI (kg/m^2^), mean ± SD	25.6 (±4.7)	25.5 (±4.4)	26.5 (±4.9)	25.5 (±4.9)	25.6 (±4.5)	25.3 (±4.9)
Clinical manifestation of tree pollen allergy, *n* (%)						
Rhinitis	1059 (99.1)	329 (99.1)	133 (98.5)	245 (98.4)	199 (99.5)	153 (100.0)
Conjunctivitis	960 (89.8)	280 (84.3)	122 (90.4)	229 (92.0)	184 (92.0)	145 (94.8)
Asthma	413 (38.6)	‐	135 (100.0)	‐	200 (100.0)	78 (51.0)
Atopic dermatitis	153 (14.3)	‐	‐	‐	‐	153 (100.0)
Other	102 (9.5)	20 (6.0)	3 (2.2)	42 (16.9)	21 (10.5)	16 (10.5)
Baseline symptoms (moderate‐to‐severe, *n* (%)						
Nasal symptoms	1021 (95.5)	320 (96.4)	129 (95.6)	234 (94.0)	191 (95.5)	147 (96.1)
Eye symptoms	833 (77.9)	238 (71.7)	100 (74.1)	201 (80.7)	170 (85.0)	124 (81.0)
Bronchial symptoms	274 (25.6)	9 (2.7%)	85 (63.0)	8 (3.2)	120 (60.0)	52 (34.0)
Skin symptoms	94 (8.8)	3 (0.9)	1 (0.7)	5 (2.0)	4 (2.0)	81 (52.9)
Pollen food syndrome, *n* (%)	558 (52.2)	‐	‐	249 (100.0)	200 (100.0)	109 (71.2)
Allergy history						
Mean age (±SD) at diagnosis of tree pollen allergy, *y*	23.7 (±14.2)	27.1 (±14.4)	28.6 (±14.7)	20.7 (±12.2)	22.1 (±14.2)	19.2 (±13.2)
Previous (completed) AIT, *n* (%)	147 (13.8)	31 (9.3)	17 (12.6)	42 (16.9)	35 (17.5)	22 (14.4)
Symptomatic medication in previous 12 months, *n* (%)						
Total symptomatic medication	1069 (100.0)	332 (100.0)	135 (100.0)	249 (100.0)	200 (100.0)	153 (100.0)
Conjunctival antihistamines	487 (45.6)	134 (40.4)	51 (37.8)	132 (53.0)	99 (49.5)	71 (46.7)
Nasal antihistamines	404 (37.8)	104 (31.3)	50 (37.0)	97 (39.0)	80 (40.0)	73 (47.7)
Oral antihistamines	897 (83.9)	262 (78.9)	96 (71.1)	232 (93.2)	174 (87.0)	133 (86.9)
Nasal corticosteroids	633 (59.2)	180 (54.2)	74 (54.8)	148 (59.4)	134 (67.0)	97 (63.4)
Inhaled corticosteroids (ICS)	264 (24.7)	8 (2.4)	87 (64.4)	13 (15.2)	107 (53.5)	49 (32.0)
Oral corticosteroids	62 (5.8)	11 (3.3)	9 (6.7)	17 (6.8)	12 (6.0)	13 (8.5)
Short‐acting ß_2_‐agonists (SABA)	216 (20.2)	21 (6.3)	65 (48.1)	8 (3.2)	86 (43.0)	36 (23.5)
Long‐acting ß_2_‐agonists (LABA)	161 (15.1)	3 (0.9)	55 (40.7)	3 (1.2)	70 (35.0)	30 (19.6)
Other	85 (8.0)	13 (3.9)	9 (6.7)	16 (6.4)	24 (12.0)	23 (15.0)
Concomitant allergies (in need of treatment)						
Grass (in need of treatment)	533 (49.9)	133 (40.1)	57 (42.2)	143 (57.4)	107 (53.5)	93 (60.8)
House dust mites (in need of treatment)	86 (8.0)	30 (9.0)	9 (6.7)	17 (6.8)	12 (6.0)	18 (11.8)
Animal hair/dander (in need of treatment)	19 (1.8)	5 (1.5)	4 (3.0)	4 (1.6)	1 (0.5)	5 (3.3)
Weed (in need of treatment)	11 (1.0)	3 (0.9)	1 (0.7)	2 (0.8)	‐	5 (3.3)
Other (in need of treatment)	7 (0.7)	1 (0.3)	‐	4 (1.6)	‐	2 (1.3)
Concomitant allergy immunotherapy (ies), *n* (%)						
All types	123 (11.5)	52 (15.7)	19 (14.1)	17 (6.8)	16 (8.0)	19 (12.4)
SLIT‐tablet(s)	110 (10.3)	44 (13.3)	19 (14.1)	15 (6.0)	17 (7.0)	18 (11.8)
SCIT	13 (1.2)	9 (2.7)	‐	‐	2 (1.0)	2 (1.3)
SLIT‐drops	4 (0.4)	1 (0.3)	1 (0.7)	2 (0.8)	‐	‐

Abbreviations: AA, allergic asthma; AD, atopic dermatitis; AIT, allergen immunotherapy; ARC, allergic rhinoconjunctivitis; ARC+AA, allergic rhinoconjunctivitis and allergic asthma; BMI, body mass index; *e*, number of events; *n*, number of patients; PFS, pollen food syndrome; SCIT, subcutaneous immunotherapy; SD, standard deviation; SLIT, sublingual immunotherapy; “‐”, no patient fulfilled the characteristic.

### 3.2 Safety

#### 3.2.1 Overall safety

A number of 2528 AEs were reported in 660 (61.7%) patients during the entire course of the study of which 2038 in 617 (57.7%) patients were treatment‐related ADRs; 1685 were local ADRs (primary endpoint) in 570 (53.3%) patients, 353 non‐local ADRs in 212 (19.8%) and 8 systemic ADRs in 7 (0.7%) patients. In patients with a history of PFS, 1232 local ADRs were reported in 379 (35.5%) patients (Table 2).

**TABLE 2:** Primary and secondary endpoints.

Primary and secondary endpoints	All patients (*n* = 1069)
	*n* (%), *e*
Treatment‐related adverse events (adverse drug reactions)	617 (57.7), 2038
Local adverse drug reactions^a^ *(primary endpoint)*	570 (53.3), 1685
Local adverse drug reactions in patients with pollen food syndrome	379 (35.5), 1232
Non‐local adverse drug reactions	212 (19.8), 353
Systemic adverse drug reactions	7 (0.7), 8
Serious adverse events	20 (1.9), 25
Serious adverse drug reactions	7 (0.7), 7

Abbreviations: *e*, number of events; *n*, number of patients.

^a^ Medical Dictionary for Regulatory Acitivities (MedDRA) Preferred Terms (PTs): lip swelling/oedema, mouth oedema, palatal oedema, swollen tongue/oedema, oropharyngeal swelling/oedema, pharyngeal oedema/pharyngeal swelling, throat tightness, laryngeal oedema.

The severity of ADRs was assessed as mild in 537 (50.2%) patients, moderate in 212 (19.8%) and severe in 50 (4.7%) patients (multiple entries). ADRs were reported most frequently at first administration and declined during the first 14 days of treatment to less than 2.5% of patients with ADRs in week 3 (see Figure S2).

ADRs were treated by medication in 151 (14.1%) patients and treatment was discontinued due to ADRs in 69 (6.5%); 25 serious AEs were reported in 20 (1.9%) patients of which 7 in 7 (0.7%) patients were ADRs. The following ADRs were documented: dyspnoea in 3 cases, angioedema, worsening of asthma, constipation, and moderate oral mucosal swelling in one case each. In no case adrenaline was administered (case narratives: Supporting Information S2). In 3 patients with dyspnoea the reactions were reported by the physician as ‘life‐threatening’ in 3 cases (1 case at first administration, 2 cases after 9 days and 4 weeks of treatment) and in 1 case, a moderate swelling of the oral mucosa at first administration was reported as serious due to ‘risk of suffocation’. Patients visited their physician and recovered in all cases with treatment by antihistamines, SABA, and corticosteroids but without adrenaline administration.

In the 5 stratified subgroups, ADRs were reported in 38.9% of patients (ARC), 42.2% (ARC + AA), 76.3% (ARC + PFS), 68.5% (ARC + AA + PFS), and 68.0% (ARC + AD (± AA ± PFS)) (Table 3).

**TABLE 3:** Patients with adverse events and adverse drug reactions.

	All patients (*n* = 1069) *n* (%), *e*	ARC (*n* = 332) *n* (%), *e*	ARC + AA (*n* = 135) *n* (%), *e*	ARC + PFS (*n* = 249) *n* (%), *e*	ARC + AA + PFS (*n* = 200) *n* (%), *e*	ARC + AD (± AA ± PFS) (*n* = 153) *n* (%), *e*
Adverse events, entire observation period	660 (61.7), 2528	141 (42.5), 374	64 (47.4), 183	199 (79.9), 815	145 (72.5), 672	111 (72.6), 484
Adverse drug reactions, entire observation period	617 (57.7), 2038	129 (38.9), 314	57 (42.2), 140	190 (76.3), 665	137 (68.5), 523	104 (68.0), 396
Mild	537 (50.2), 1478	116 (34.9), 240	48 (35.6), 114	162 (65.1), 474	121 (60.5), 376	90 (58.8), 274
Moderate	212 (19.8), 478	30 (9.0), 62	20 (14.8), 24	69 (27.7), 161	49 (24.5), 124	44 (28.8), 107
Severe	50 (4.7), 81	6 (1.8), 12	2 (1.5), 2	18 (7.2), 30	15 (7.5), 23	9 (5.9), 14
Serious	7 (0.7), 7	‐	2 (1.5), 2	‐	4 (2.0), 4	1 (0.7), 1
Treated by medication	151 (14.1), 291	21 (6.3), 41	9 (6.7), 12	54 (21.7), 105	35 (17.5), 81	32 (20.9), 521
Discontinued	69 (6.5), 132	12 (3.6), 24	5 (3.7), 9	22 (8.8), 45	16 (8.0), 28	14 (9.2), 26
Adverse events, first administration	499 (46.7), 995	106 (31.9), 169	43 (31.9), 81	153 (61.4), 311	118 (59.0), 249	79 (51.6), 185
Adverse drug reactions, first administration	491 (45.9), 973	105 (31.6), 167	42 (31.1), 80	150 (60.2), 302	117 (58.5), 243	77 (50.3), 181
Mild	437 (40.9), 763	97 (29.2), 144	40 (29.6), 71	132 (53.0), 237	101 (50.5), 182	67 (43.8), 129
Moderate	103 (9.6), 189	14 (4.2), 20	6 (4.4), 8	35 (14.1), 60	25 (12.5), 53	23 (15.0), 48
Severe	15 (1.4), 21	1 (0.3), 3	1 (0.7), 1	4 (1.6), 5	6 (3.0), 8	3 (2.0), 4
Serious	3 (0.3), 3	‐	‐	‐	2 (1.0), 2	1 (0.7), 1
Treated by medication	46 (4.3), 82	11 (3.3), 16	3 (2.2), 5	17 (6.8), 32	8 (4.0), 18	7 (4.6), 11
Discontinued	19 (1.8), 27	4 (1.2), 5	1 (0.7), 1	6 (2.4), 9	4 (2.0), 4	4 (2.6), 8

Abbreviations: AA, allergic asthma; AD, atopic dermatitis; ARC, allergic rhinoconjunctivitis; ARC+AA, allergic rhinoconjunctivitis and allergic asthma; *e*, number of events; *n*, number of patients; PFS, pollen food syndrome.

**FIGURE 1:**
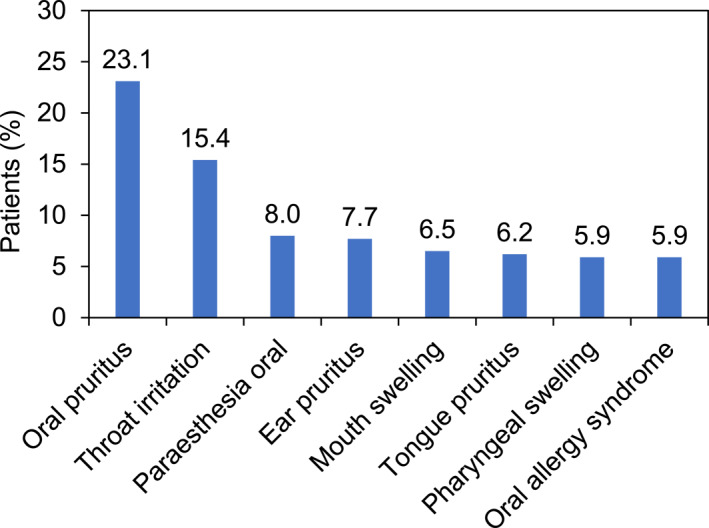
Most frequent adverse drug reactions (≥5% of total patients) during the entire course of the study.

Most frequent MedDRA PTs were oral pruritus in 247 (23.1%) patients, throat irritation in 165 (15.4%) patients, paraesthesia oral in 86 (8.0%) and ear pruritus in 82 (7.7%) patients (Figure 1).

#### 3.2.2 Tolerability at first administration

ADRs at first administration of the SQ tree SLIT‐tablet were reported in 491 (45.9%) patients, assessed as mild in 437 (40.9%), moderate in 103 (9.6%) and severe in 15 (1.4%) patients (multiple entries), and were treated by medication in 46 (4.3%). ADRs were classified as serious in 3 (0.3%) patients; 19 (1.8%) patients discontinued treatment due to ADRs.

Frequencies of ADRs (≥1% of patients) as MedDRA SOCs and PTs in total patients and the 5 subgroups are available in Table ST1.

#### 3.2.3 Initiation of treatment during pollen exposure

In 192 (18.0%) patients, physicians recorded current pollen exposure with tree pollen at initiation of treatment. The proportion of patients with ADRs was not increased versus patients without exposure (28.1% vs. 49.8%) (Table ST2).

#### 3.2.4 Coadministration with other SLIT‐tablets

Additional SLIT was initiated in 473/1021 (46.3%) patients (one SLIT: 442 (43.3%), two: 30 (2.9%), 1 missing; 438 grass‐, 57 house dust mite (HDM)‐tablets). In 335/1021 patients the additional SLIT was first administered during any follow‐up visit; 303/335 (90.4%) received the SQ grass tablet initially at visit 2. The mean interval between the administration of the two SLIT‐tablets with different allergens was 265.2 (±353.9) minutes (median 10.0). The number of severe AEs was slightly higher with coadministration of two tablets (35/447 (7.8%) patients, *e* = 67) versus sole administration of the tree tablet (36/622 (5.8%), *e* = 56), but the number of serious AEs was lower (4 (0.9%), *e* = 5 versus 16 (2.6%), *e* = 20); treatment was discontinued due to AEs in 19 (5.2%) patients with two and in 65 (21.8%) with one tablet (Table ST3). In patients with coadministration of SQ tree‐ and SQ grass‐tablets, the proportion of patients with AEs was 1.4‐fold higher than for treatment with the tree tablet alone (two tablets: 335/454 (73.8%) patients, *e* = 1453; one tablet: 325/615 (52.8%), *e* = 1075).

### 3.3 Effectiveness

#### 3.3.1 Symptoms and use of medication

**FIGURE 2:**
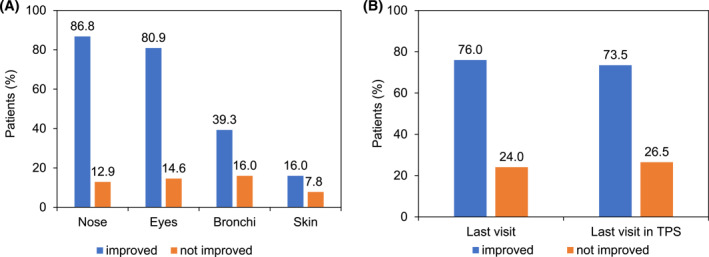
(A) Change in symptoms (improved: no symptoms or symptoms decreased; not improved: symptoms unchanged or increased) at the individual last visit versus baseline (all patients, *n* = 1021; difference to 100%: patients not affected), (B) change in use of symptomatic medication (improved: no symptomatic medication or medication decreased; not improved: symptomatic medication unchanged or increased) at the individual last visit (all patients, *n* = 1021) and at the individual last visit within the estimated tree pollen season (TPS), (*n* = 702 patients), versus baseline. Periods of the tree pollen seasons were estimated for Germany, Denmark, and The Netherlands as: February to June 2021, January to June 2022, and Finland, Norway, Sweden as: March to July 2021, February to July 2022.

The proportions of patients whose symptoms improved (no symptoms/symptoms decreased) at the individual last visit versus baseline (difference to 100%: patients not affected) are displayed in Figure 2A. In most patients with available data (*n* = 1021) symptoms improved (nose: 86.9%; eyes: 80.9%, bronchi: 39.3%, skin: 16.1%). Results were very similar if this analysis was restricted to patients who had their individual last visit within the estimated period of the tree pollen season with potential exposure to tree pollen (Figure S3).

The proportions of patients using symptomatic medication are displayed in Figure 2B. The use of symptomatic medication had improved (no symptomatic medication/medication decreased) at the individual last visit versus baseline (75.9%). Restricting the analysis to patients who had their last visit within the estimated period of tree pollen exposure (*n* = 702), revealed similar proportions of patients with improvement in the use of symptomatic medication (73.5%).

#### 3.3.2 Pollen food syndrome

**FIGURE 3:**
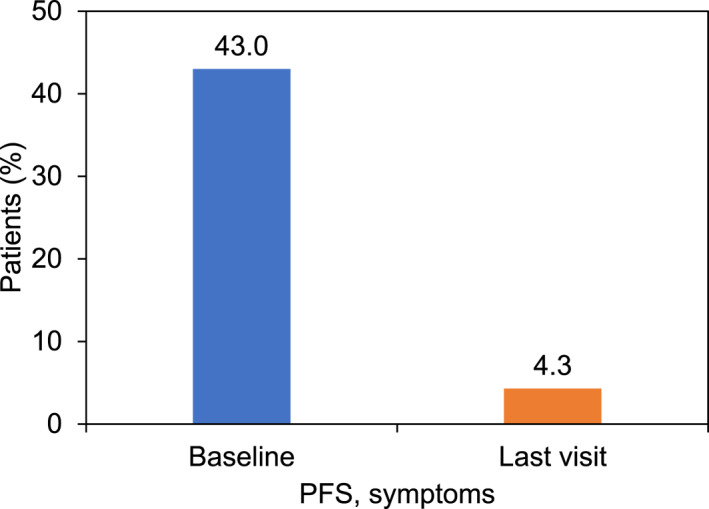
Proportion of patients with pollen food syndrome (PFS) at baseline (*n* = 1069) and at the individual last visit of the study (*n* = 1021).

The proportions of patients who had PFS with symptoms at baseline and at the end of the study are displayed in Figure 3. ‘PFS, no symptoms’ was recorded in 9.3% of total patients at baseline and in 50.0% at the individual last visit.

#### 3.3.3 Adherence

The average frequency of taking the tablet since the last visit was recorded by the physicians as 6 to 7 times per week in 965 (94.5%) of 1021 patients with follow‐up visits, 4 to 5 times in 43 (4.2%), and ≤3 times in 13 (1.3%). The most frequent reasons for not taking the tablet every day were ‘forgotten’ (61.3% of patients) and ‘due to AE’ (17.5%). Treatment was continued by 896 (83.8%) patients at the end of the study.

## 4 DISCUSSION

In this prospective, multi‐national NIS‐PASS, treatment with the SQ tree SLIT‐tablet in >1000 adult patients in the real‐life setting was analysed.

During the entire course of the study, local ADRs were reported in 53.3% of patients, non‐local ADRs in 19.8% of patients, and systemic ADRs in 0.7% of patients; 7 ADRs were assessed as serious. Treatment was discontinued due to ADRs in 6.5% of patients. All patients with ADRs reported as serious cases recovered without the administration of adrenaline. According to pooled data of all subjects from 48 timothy grass, ragweed, house dust mite and tree SLIT‐tablet trials, anaphylaxis was rare for SLIT‐tablets.^17^

In 3 cases dyspnoea was reported verbatim as ‘life‐threatening’ by the physician (1 case at first administration, 2 cases after 9 days and 4 weeks of treatment initiation) and in 1 case a moderate swelling of the oral mucosa at first administration of the tablet was reported as serious by the physician due to ‘risk of suffocation’. Patients visited their physician and recovered in all cases with treatment by antihistamines, SABA, and corticosteroids but without adrenaline. Taken together, the known good safety profile of SLIT‐tablets allowing at home treatment is not considered to be compromised by these cases. However, a well‐informed and ‐educated patient on the treatment‐principles and potential side‐effects of AIT should always be a prerequisite for AIT in general.^18^

The analysis of subgroups of patients (ARC with/without AA, and with/without PFS) revealed that the proportions of patients with ADRs during the entire observation period were similar in patients with ARC and patients with ARC and AA but increased if patients had PFS. Thus, PFS may potentially be considered as a risk factor for a higher frequency and severity of ADRs. The frequency of ADRs was similar or lower compared with data of the trial TT‐04, in which AEs were reported in 82% of patients, and oral pruritus (36%) and throat irritation (23%) were the most common reactions.^12^

Overall, this NIS‐PASS with the SQ tree SLIT‐tablet confirms the safety and tolerability profile known from the clinical trials of the clinical development program.^9^, ^10^, ^11^, ^12^

In the group of patients exposed to tree pollen at the start of treatment with the SQ tree SLIT‐tablet, ADRs were not observed to increase compared with patients with the start of treatment outside the tree pollen season, indicating that the safety and tolerability at the initiation of treatment with the SQ tree SLIT‐tablet is not impaired by exposure to tree pollen, as previously demonstrated in clinical trials with sublingual and subcutaneous AIT in subjects with grass pollen allergy.^19^, ^20^

A higher proportion of patients with AEs and number of AEs (1.4‐fold) was observed with coadministration of two SQ SLIT‐tablets (grass and trees) than with only one tablet (tree), probably because poly‐allergic patients may be more affected by their allergy. However, the number of AEs did not double as may be expected for a treatment with two tablets, in line with data published for the combination of other SQ SLIT‐tablets (grass and ragweed).^15^ Fewer patients discontinued treatment with coadministration of two SLIT‐tablets versus single administration of the tree tablet, possibly because patients who require treatment with two SLIT‐tablets experience a higher burden of disease^21^ and are thus more motivated to adhere to treatment.

Evaluating effectiveness of treatment with the SQ tree SLIT‐tablet, symptoms had improved and use of symptomatic medication decreased at the last visit of the study versus baseline (i.e. tree pollen season prior to AIT) in line with the efficacy demonstrated in the trial TT‐04.^12^ No considerable differences were obtained if the change of symptoms and use of symptomatic medication was analysed in all patients or in a subgroup of patients who had their individual last visit during estimated periods of potential exposure to tree pollen in central and northern Europe.

Comparing the status of PFS at the last visit of the study with baseline assessments for the previous 12 months before initiation of AIT, 4.3% of patients had PFS with symptoms at the individual last visit compared with 43.0% at baseline. This marked decrease may suggest a positive effect of treatment with the SQ tree SLIT‐tablet on symptoms of PFS, although the observation periods for baseline assessment and individual last visit were different (last 12 months before initiation of AIT at baseline vs. 1–3 months for the individual last visit). However, patients with PFS with symptoms are likely avoiding foods that trigger their PFS and may thus be recorded as ‘PFS, no symptoms’ during follow‐up. An improvement of PFS was previously reported after a controlled food challenge in a subgroup of patients with PFS to apple performed at the end of trial TT‐04.^22^ The European Academy of Allergy and Clinical Immunology (EAACI) position paper on PFS requires further clinical trials to assess the effect of birch AIT on PFS.^23^

Continuation of treatment with the tree tablet after the average 5.4‐months observation period of our study was recorded in 83.8% of patients. In retrospective analyses of prescription data bases, persistence rates of 41% after two years of treatment with the SQ grass SLIT‐tablet,^24^ and 29.5%–36.5% with two grass SLIT‐tablets were reported.^25^ In a prospective study with the SQ grass SLIT‐tablet, 67.4% of patients continued treatment after an average treatment period of 1 year.^26^

Limitations of our study are related to the open‐label, uncontrolled, observational design. Safety data were reported, following the procedures for spontaneous case reporting during routine treatment with the SQ tree SLIT‐tablet. The frequency of AE‐reporting is expected to be higher in a cohort of patients included in a NIS‐PASS focussing on safety and tolerability than in patients routinely treated without being included in a study.

Data on effectiveness are limited in a NIS‐PASS due to the primary focus on safety.^27^ Due to the lack of a control group, changes in symptoms and symptomatic medication use were related to the assessments prior to the start of AIT (previous tree pollen season, previous symptoms of PFS). Symptoms and use of symptomatic medication may have been influenced by the individual tree pollen exposure of the patients. The start and duration of the tree pollen season were different between countries and regions and treatment with the SQ tree SLIT‐tablet was initiated within the tree pollen season or at different time points thereafter. A study using propensity score matching of treatment groups has recently demonstrated the effectiveness of AIT in real‐life by a design with low risk of bias.^28^, ^29^

As first of its kind, we, here, report first data on safety and tolerability of the SQ tree SLIT‐tablet under real‐life conditions.

## 5 CONCLUSION

In conclusion, the results from this prospective non‐interventional post‐authorisation safety study confirm the safety and tolerability profile of the SQ tree SLIT‐tablet known from placebo‐controlled clinical trials. Data indicate that a start of treatment within the tree pollen season or coadministration of two SLIT‐tablets do not impair safety. The number and proportions of patients with local ADRs were higher in patients with PFS. Data on symptoms and use of medication at the last study visit versus baseline assessments support the clinical effectiveness of treatment in real‐life in line with published data on clinical efficacy from placebo‐controlled clinical trials. PFS with symptoms declined comparing assessments at the last visit and baseline, which may be attributed to avoiding the food triggering PFS and/or to an effect of AIT.

## AUTHOR CONTRIBUTIONS

**Oliver Pfaar**: Investigation; writing‐review & editing. **Hendrik Wolf**: Conceptualisation; methodology; project administration; supervision; writing‐original draft; writing‐review & editing. **Rainer Reiber**: Investigation; writing‐review & editing. **André Knulst**: Investigation; writing‐review & editing. **Kirsten Sidenius**: Investigation; writing‐review & editing. **Mika Mäkelä**: Investigation; writing‐review & editing. **Sverre Steinsvåg:** Investigation; writing‐review & editing. **Christer Janson**: Investigation; writing‐review & editing. **Leonard van der Zwan**: Methodology; project administration; supervision; writing‐review & editing. **Elena Uss**: Conceptualisation; methodology; project administration; supervision; writing‐review & editing. **Peter Arvidsson**: Conceptualisation; methodology; project administration; supervision; writing‐review & editing. **Kathrin Borchert**: Data curation; formal analysis; project administration; supervision; writing‐review & editing. **Helena Himmelhaus**: Data curation; formal analysis; project administration; supervision; writing‐review & editing. **Eike Wüstenberg**: Conceptualisation; methodology; supervision; writing‐review & editing.

## CONFLICT OF INTEREST STATEMENT

Oliver Pfaar reports grants fees for the trial reported. In addition, he reports grants and/or personal fees and/or travel support from ALK‐Abelló, Allergopharma, Stallergenes Greer, HAL Allergy Holding B.V./HAL Allergie GmbH, Bencard Allergie GmbH/Allergy Therapeutics, Lofarma, ASIT Biotech Tools S.A., Laboratorios LETI/LETI Pharma, GlaxoSmithKline, ROXALL Medizin, Novartis, Sanofi‐Aventis and Sanofi‐Genzyme, Med Update Europe GmbH, streamedup! GmbH, Pohl‐Boskamp, Inmunotek S.L John Wiley and Sons, AS, Paul‐Martini‐Stiftung (PMS), Regeneron Pharmaceuticals Inc., RG Aerztefortbildung, Institut für Disease Management, Springer GmbH, AstraZeneca, IQVIA Commercial, Ingress Health, Wort&Bild Verlag, Verlag ME, Procter&Gamble, ALTAMIRA, Meinhardt Congress GmbH, Deutsche Forschungsgemeinschaft, Thieme, Deutsche AllergieLiga e.V., AeDA, Alfried‐Krupp Krankenhaus, Red Maple Trials Inc., Königlich Dänisches Generalkonsulat, Medizinische Hochschule Hannover, ECM Expro&Conference Management, Technical University Dresden, EAACI, Lilly, Paul‐Ehrlich Institut, Almirall, all outside the applied TF‐activity. He is member of EAACI Excom, member of ext. board of directors DGAKI; coordinator, main‐ or co‐author of different position papers and guidelines in rhinology, allergology and allergen‐immunotherapy. Christer Janson has received honoraria for educational activities and lectures from AstraZeneca, ALK, Chiesi, GlaxoSmithKline, Novartis, Orion, and Sanofi, and has served on advisory boards arranged by AstraZeneca, GlaxoSmithKline, Novartis, Orion and Sanofi. Hendrik Wolf, Eike Wüstenberg, Leonard van der Zwan, Elena Uss and Peter Arvidsson are employees of ALK, HW and EW hold stock options of ALK. Kathrin Borchert and Helena Himmelhaus are employees of Xcenda GmbH, part of Cencora, a company which received funding from ALK to conduct the study. All authors had full access to all the data in this study and take complete responsibility for the integrity of the data and accuracy of the data analysis. Rainer Reiber, André Knulst, Kirsten Sidenius, Mika Mäkelä and Sverre Steinsvåg declared no conflicts of interest.

## Supporting information

Supporting Information S1

## Data Availability

The data that support the findings of this study are available from the corresponding author upon reasonable request.
