# Supplementary material for: Treatment with the SQ tree sublingual immunotherapy tablet is safe and well tolerated in real‐life
Source: Clin Transl Allergy. 2024 Jul 2;14(7):e12373. doi: 10.1002/clt2.12373 (PMC11219271; doi:10.1002/clt2.12373)
Supplement: Supplementary file 1 — Supporting Information S1 [file CLT2-14-e12373-s001.docx]

***Supporting information S1***

**Approval of the study by responsible ethics committees** **and national authorities**

*Germany:* Paul-Ehrlich-Institut, Landesärztekammer Baden-Württemberg, Reference No. F-2019-104; University of Marburg, “Studie 200/19”; University of Jena, “2020-1688-AWB”; *Denmark:* NI-studies are not reviewed by ethics committees, *Finland:* Ethics Committee IV, Reference number HUS/3257/2019; *The Netherlands:* De Adviescommissie nWMO Martini Ziekenhuis Groningen, Reference No. nWMO020.01.001; *Norway:* Regional Committees for Medical and Health Research Ethics, Reference number 60025; *Sweden:* Ethics Review Board Uppsala, Reference number 2019-05865.

***Supporting information S2***

**Case narratives of serious ADRs**

***Case 1:***

A 29-year-old female patient with ARC and AA to tree pollen and concomitant allergies to grasses and rye, house dust mites, and animal hair and dander with PFS, Crohn’s disease and eczema developed dyspnoea and itching in the mouth, at first administration of ITULAZAX^®^. The dyspnoea was reported as life-threatening by the physician. Five minutes after onset, the events were treated with oral antihistamine and inhaled ß_2_-agonist and the patient recovered. The patient reported flu-like symptoms on the same day that were not treated and recovered after 6 days. Treatment with ITULAZAX^®^ was continued with reduced dose (1/8 tablet). Five minutes after administration of ITULAZAX^®^ the patient reported a feeling of swelling in the throat that was not observed by the physician, dyspnoea and itching in the mouth. Treatment with ITULAZAX^®^ was discontinued and the patient recovered on the same day.

***Case 2:***

A 32-year-old female patient with ARC and AA to tree and concomitant allergies to grass/rye, house dust mites, animal hair and dander with eczema experienced a tingling tongue and throat, at the same day of first administration of ITULAZAX^®^ which was not treated by medication. Treatment with ITULAZAX^®^ was continued. About 4 weeks after first administration of ITULAZAX^®^, the patient had a feeling of a flu and experienced acute dyspnoea 6 hours after administration of ITULAZAX^®^ that was considered as life-threatening by the physician, chest pain, and hoarseness (x-ray of the chest without abnormalities). The events were treated with inhaled ß2-agonist and inhaled and oral corticosteroids. Treatment with ITULAZAX^®^ was discontinued. On the following day the patient was considered recovered from tingling tongue and throat, acute dyspnoea, chest pain and feeling of a flu, and recovered from hoarseness, about 2 weeks later.

***Case 3:***

A 41-year-old female with allergy to tree pollen and animal hair and dander, and allergic rhinitis, asthma, PFS, and depression started to experience constipation and general feeling of unwellness, and nausea after first administration with ITULAZAX^®^. The patient was treated by a histamine antagonist. After about two weeks of treatment with ITULAZAX^®^ the patient started coughing a lot with moderate intensity and recovered after treatment with a bronchodilator. Six days later the patient experienced a moderately itching tongue when eating tomato which was coded as PT oral allergy syndrome and condition aggravated by the marketing authorisation holder. About two and a half months later the patient recovered with sequelae from constipation. The event was assessed by the physician as serious because it resulted in persistent or significant disability or incapacity. The patient recovered from constipation, general feeling of unwellness and nausea, but not from having an itchy tongue when eating tomato and coughing. Treatment with ITULAZAX^®^ was continued without change. One week later, the patient withdrew from treatment due to constipation.

***Case 4:***

A 26-year-old female patient with ARC and AA to tree pollen and concomitant allergies to animal hair and dander with PFS and food allergy experienced itching of the mucosa 5 minutes after first administration of ITULAZAX^®^ and recovered spontaneously without treatment. On day 9 of treatment, the patient experienced rhinitis, feeling of swollen throat and swollen eyes, 5 minutes after administration of ITULAZAX^®^, and after 5 to 10 minutes dyspnoea that was reported by the physician as life-threatening. The patient was treated by oral antihistamine and long-acting ß_2_-agonist and recovered. Two days later, the dose of ITULAZAX^®^ was reduced to half a tablet per day and the treatment with ITULAZAX^®^ was continued.

***Case 5:***

A 49-year-old male patient with ARC and AA to tree pollen experienced mild tingling of the mouth and tingling under the tongue immediately after first administration of ITULAZAX^®^ and recovered without treatment after 20 minutes. After about 20 days, the patient experienced a swollen uvula, 13 hours after administration of the tablet that was diagnosed as spontaneous angioedema. The event was reported as medically significant and, thus, serious by the physician. The patient recovered after treatment with oral antihistamine, about 6 days later. The dose of ITULAZAX^®^ was reduced to half a tablet.

***Case 6:***

A 48-year-old female patient with ARC and AA to tree pollen and grass/rye, with PFS experienced itching mouth, itching throat, and itching ears immediately after first administration of ITULAZAX^®^ and recovered 6 days later without treatment. About 7 days after first administration of ITULAZAX^®^, the patient experienced an asthma exacerbation. The patient was hospitalized due to worsening of asthma for 5 days and treated with fenoterol hydrobromide, prednisolone (30 minutes after onset), and beclomethasone, treatment with ITULAZAX^®^ was discontinued. The patient recovered from the asthma exacerbation, about 8 days after treatment. Prior to worsening of asthma, the patient and her husband had a small case of catharalia; no infection was found. During the hospital admission, the asthma treatment was corrected, and the patient was found to have moderate dysfunctional breathing, and, probably, also mild-to-moderate inspiratory laryngeal obstruction. In the period prior to worsening of asthma and hospital admission, the patient had been under great stress.

***Case 7:***

A 41-year-old female with ARC, AA, and atopic dermatitis to tree pollen and concomitant allergies to grass pollen, with PFS experienced a moderate swelling of the oral mucosa immediately after first administration of ITULAZAX^®^ treated by oral antihistamine and recovered after 3 hours. The event was assessed as serious by the physician due to risk of suffocation and treatment with the ITULAZAX^®^ was discontinued.

**Table ST1:** Patients with adverse drug reactions in ≥1% of patients in any of the groups

| *MedDRA System Organ Class*  MedDRA Preferred Term | All patients  (n=1069)  n (%), e | ARC  (n=332)  n (%), e | ARC+AA  (n=135)  n (%), e | ARC+PFS  (n=249)  n (%), e | ARC+AA+PFS  (n=200)  n (%), e | ARC+AD±AA±PFS  (n=153)  n (%), e |
| --- | --- | --- | --- | --- | --- | --- |
| All patients with adverse drug reactions | 617 (57.7), 2038 | 129 (38.9), 314 | 57 (42.2), 140 | 190 (76.3), 665 | 137 (68.5), 523 | 104 (68.0), 396 |
| *Ear and labyrinth disorders* | *94 (8.8), 111* | *15 (4.5), 19* | *10 (7.4), 15* | *36 (14.5), 39* | *16 (8.0), 18* | *17 (11.1), 20* |
| Ear pruritus | 82 (7.7), 94 | 12 (3.6), 15 | 9 (6.7), 13 | 30 (12.0), 32 | 16 (8.0), 18 | 15 (9.8), 16 |
| *Eye disorders* | 26 (2.4), 75 | 4 (1.2), 4 | 3 (2.3), 3 | 9 (3.6), 17 | 4 (2.0), 41 | 6 (3.9), 10 |
| Eye pruritus | 16 (1.5), 28 | 3 (0.9), 3 | 2 (1.5), 2 | 5 (2.0), 5 | 3 (1.5), 15 | 3 (2.0), 3 |
| *Gastrointestinal disorders* | *473 (44.2), 998* | *95 (28.6), 175* | *42 (31.1), 64* | *146 (58.6), 321* | *113 (56.5), 252* | *77 (50.3), 186* |
| Dyspepsia | 15 (1.4), 20 | 1 (0.3), 1 | 1 (0.7), 1 | 8 (3.2), 10 | 2 (1.0), 4 | 3 (2.0), 4 |
| Dysphagia | 12 (1.1), 12 | 2 (0.6), 2 | 1 (0.7), 1 | - | 5 (2.5), 5 | 4 (2.6), 4 |
| Gastrooesophageal reflux disease | 13 (1.2), 13 | - | - | 5 (2.0), 5 | 4 (2.0), 4 | 4 (2.6), 4 |
| Hypoaesthesia oral | 16 (1.5), 18 | 8 (2.4), 9 | 1 (0.7), 2 | 3 (1.2), 3 | 1 (0.5), 1 | 3 (2.0), 4 |
| Lip pruritus | 29 (2.7), 36 | 3 (0.9), 4 | 1 (0.7), 1 | 10 (4.0), 10 | 11 (5.5), 17 | 4 (2.6), 4 |
| Lip swelling | 39 (3.6), 50 | 5 (1.5), 6 | 4 (3.0), 7 | 13 (5.2), 15 | 9 (4.5), 10 | 8 (5.2), 12 |
| Mouth swelling | 70 (6.5), 83 | 11 (3.3), 18 | 7 (5.2), 7 | 25 (10.0), 26 | 12 (6.0), 13 | 15 (9.8), 19 |
| Nausea | 15 (1.4), 16 | 1 (0.3), 1 | - | 7 (2.8), 7 | 2 (1.0), 2 | 5 (3.3), 6 |
| Oral discomfort | 13 (1.2), 18 | 1 (0.3), 1 | 4 (3.0), 5 | 3 (1.2), 3 | 3 (1.5), 4 | 2 (1.3), 5 |
| Oral mucosal blistering | 14 (1.3), 14 | 2 (0.6), 2 | 1 (0.7), 1 | 6 (2.4), 6 | 4 (2.0), 4 | 1 (0.7), 1 |
| Oral pruritus | 247 (23.1), 326 | 36 (10.8), 49 | 20 (14.8), 22 | 79 (31.7), 99 | 76 (38.0), 108 | 36 (23.5), 48 |
| Paraesthesia oral | 86 (8.0), 135 | 31 (9.3),43 | 4 (3.0), 5 | 24 (9.6), 53 | 16(8.0), 19 | 11 (7.2), 15 |
| Swollen tongue | 41 (3.8), 42 | 6 (1.8), 6 | 1 (0.7), 1 | 16 (6.4), 16 | 10 (5.0), 10 | 8 (5.2), 9 |
| Tongue pruritus | 66 (6.2), 93 | 8 (2.4), 9 | 6 (4.4), 7 | 20 (8.0), 26 | 19 (9.5), 24 | 13 (8.5), 27 |
| *General disorders and administration site conditions* | *113 (10.6), 158* | *11 (3.3), 12* | *5 (3.7), 7* | *41 (16.5), 59* | *30 (15.0), 42* | *26 (17.0), 38* |
| Condition aggravated | 56 (5.2), 76 | 2 (0.6), 2 | - | 27 (10.8), 33 | 15 (7.5), 20 | 12 (7.8), 21 |
| Fatigue | 21 (2.0), 22 | 2 (0.6), 2 | 1 (0.7), 1 | 7 (2.8), 7 | 7 (3.5), 8 | 4 (2.6), 4 |
| Sensation of foreign body | 30 (2.8), 31 | 4 (1.2), 5 | 1 (0.7), 1 | 11 (4.4), 11 | 8 (4.0), 8 | 6 (3.9), 6 |
| *Immune system* | *67 (6.3), 92* | *5 (1.5), 7* | *2 (1.5), 3* | *27 (10.8), 37* | *17 (8.5), 21* | *16 (10.5), 24* |
| Oral allergy syndrome | 63 (5.9), 86 | 5 (1.5), 7 | 2 (1.5), 3 | 25 (10.0), 33 | 16 (8.0), 20 | 15 (9.8), 23 |
| *Injury, poisoning and procedural complications* | *30 (2.8), 31* | *11 (3.3), 11* | *2 (1.5), 2* | *7 (2.8), 8* | *5 (2.5), 5* | *5 (3.3), 5* |
| Intentional underdose | 15 (1.4), 15 | 4 (1.2), 4 | 1 (0.7), 1 | 3 (1.2), 3 | 4 (2.0), 4 | 3 (2.0), 3 |
| Off-label use | 27 (2.5), 28 | 11 (3.3), 11 | 2 (1.5), 2 | 5 (2.0), 6 | 4 (2.0), 4 | 5 (3.3), 5 |
| Product administered at inappropriate site | 11 (1.0), 11 | 7 (2.1), 7 | 1 (0.7), 1 | 2 (0.8), 2 | - | 1 (0.7), 1 |
| Wrong technique in product usage process | 17 (1.6), 17 | 4 (1.2), 4 | 1 (0.7), 1 | 4 (1.6), 4 | 4 (2.0), 4 | 4 (2.6), 4 |
| *Nervous system disorders* | *18 (1.7), 33* | *3 (0.9), 3* | *4 (3.0), 4* | *5 (2.0), 16* | *1 (0.5), 1* | *5 (3.3), 9* |
| Headache | 6 (0.6), 7 | 1 (0.3), 1 | 3 (2.2), 3 | 1 (0.4), 2 | 1 (0.5), 1 | - |
| Paraesthesia | 6 (0.6), 18 | - | - | 3 (1.2), 12 | - | 3 (2.0), 6 |
| *Respiratory, thoracic, and mediastinal disorders* | *312 (29.2), 533* | *51 (15.4), 76* | *23 (17.0), 37* | *106 (42.6), 177* | *73 (36.5), 144* | *59 (38.6), 99* |
| Asthma | 11 (1.0), 12 | 1 (0.3), 1 | 1 (0.7), 1 | 1 (0.4), 1 | 8 (4.0), 9 | - |
| Cough | 24 (2.2), 27 | 2 (0.6), 3 | 4 (3.0), 4 | 11 (4.4), 12 | 3 (1.5), 4 | 4 (2.6), 4 |
| Dry throat | 12 (1.1), 12 | 3 (0.9), 3 | 1 (0.7), 1 | 2 (0.8), 2 | 4 (2.0), 4 | 2 (1.3), 2 |
| Dyspnoea | 26 (2.4), 36 | 3 (0.9), 3 | 2 (1.5), 2 | 7 (2.8), 9 | 9 (4.5), 16 | 5 (3.3), 6 |
| Oropharyngeal pain | 23 (2.2), 25 | 5 (1.5), 7 | 2 (1.5), 2 | 12 (4.8), 12 | 4 (2.0), 4 | - |
| Pharyngeal paraesthesia | 14 (1.3), 17 | 4 (1.2), 5 | 1 (0.7),1 | 5 (2.0), 6 | 3 (1.5), 3 | 1 (0.7), 2 |
| Pharyngeal swelling | 63 (5.9), 69 | 12 (3.6), 13 | 6 (4.4), 6 | 16 (6.4), 18 | 14 (7.0), 16 | 15 (9.8), 16 |
| Rhinorrhoea | 12 (1.1), 33 | 4 (1.2), 4 | - | 4 (1.6), 6 | 3 (1.5), 20 | 1 (0.7), 3 |
| Sneezing | 13 (1.2), 13 | 4 (1.2), 4 | - | 4 (1.6), 4 | 2 (1.0), 2 | 3 (2.0), 3 |
| Throat irritation | 165 (15.4), 205 | 24 (7.2), 27 | 16 (11.9), 18 | 56 (22.5), 70 | 33 (16.5), 41 | 36 (23.5), 49 |
| Throat tightness | 11 (1.0), 13 | 1 (0.3), 1 | - | 5 (2.0), 5 | 5 (2.5), 7 | - |
| *Skin disorders* | *49 (4.6), 63* | *5 (1.5), 6* | *4 (3.0), 4* | *17 (6.8), 21* | *9 (4.5), 11* | *14 (9.2), 21* |
| Pruritus | 20 (1.9), 23 | 2 (0.6), 3 | 3 (2.2), 3 | 5 (2.0), 6 | 3 (1.5), 3 | 7 (4.6), 8 |

*AA:* allergic asthma, *AD:* atopic dermatitis, *ADR:* adverse drug reaction, *ARC:* allergic rhinoconjunctivitis*, ARC+AA:* allergic rhinoconjunctivitis and allergic asthma, *PFS:* pollen food syndrome, *n:* number of events, *e: number of* events, *MedDRA*: Medical Dictionary for Drug Regulatory activities

**Table ST2:** Adverse drug reactions at first administration of the SQ tree SLIT-tablet during tree pollen exposure vs no exposure

|  | Exposure to tree pollen (n=192)  n^†^ (%), e | No exposure to tree pollen (n=877)  n^†^ (%), e |
| --- | --- | --- |
| All adverse drug reactions at first administration | 54 (28.1), 73 | 437 (49.8), 900 |
| mild | 48 (25.0), 60 | 389 (44.4), 703 |
| moderate | 10 (5.2), 12 | 93 (10.6), 177 |
| severe | 1 (0.5), 1 | 14 (1.6), 20 |
| serious | 1 (0.5), 1 | 2 (0.2), 2 |
| treated by medication | 5 (2.6), 8 | 41 (4.7), 74 |
| discontinued | 3 (1.6), 6 | 16 (1.8), 21 |

*ADR:* adverse drug reaction, *n:* number of patients*, e:* number of events,

^†^double counting of patients possible as one patient could have more events with different characteristics regarding causality, severity etc.

**Table ST3:** Adverse events with coadministration of different SLIT-tablets vs single administration of the SQ tree SLIT-tablet during the entire course of treatment

|  | Coadministration of SLIT-tablets (n=447)  n^†^ (%), e | Only SQ tree SLIT-tablet (n=622)  n^†^ (%), e |
| --- | --- | --- |
| All adverse events | 362 (81.0), 1531 | 298 (47.9), 997 |
| mild | 326 (72.9), 1149 | 246 (39.5), 653 |
| moderate | 141 (31.5), 315 | 114 (18.3), 287 |
| severe | 35 (7.8), 67 | 36 (5.8), 56 |
| serious | 4 (0.9), 5 | 16 (2.6), 20 |
| treated by medication | 110 (24.6), 216 | 96 (15.4), 181 |
| discontinued | 19 (4.3), 36 | 65 (10.5), 123 |

*ADR:* adverse drug reaction, *n:* number of patients*, e:* number of events,

^†^double-counting of patients possible as one patient could have more events with different characteristics regarding causality, severity etc.

**Figure S1.** Flow of patients through the study


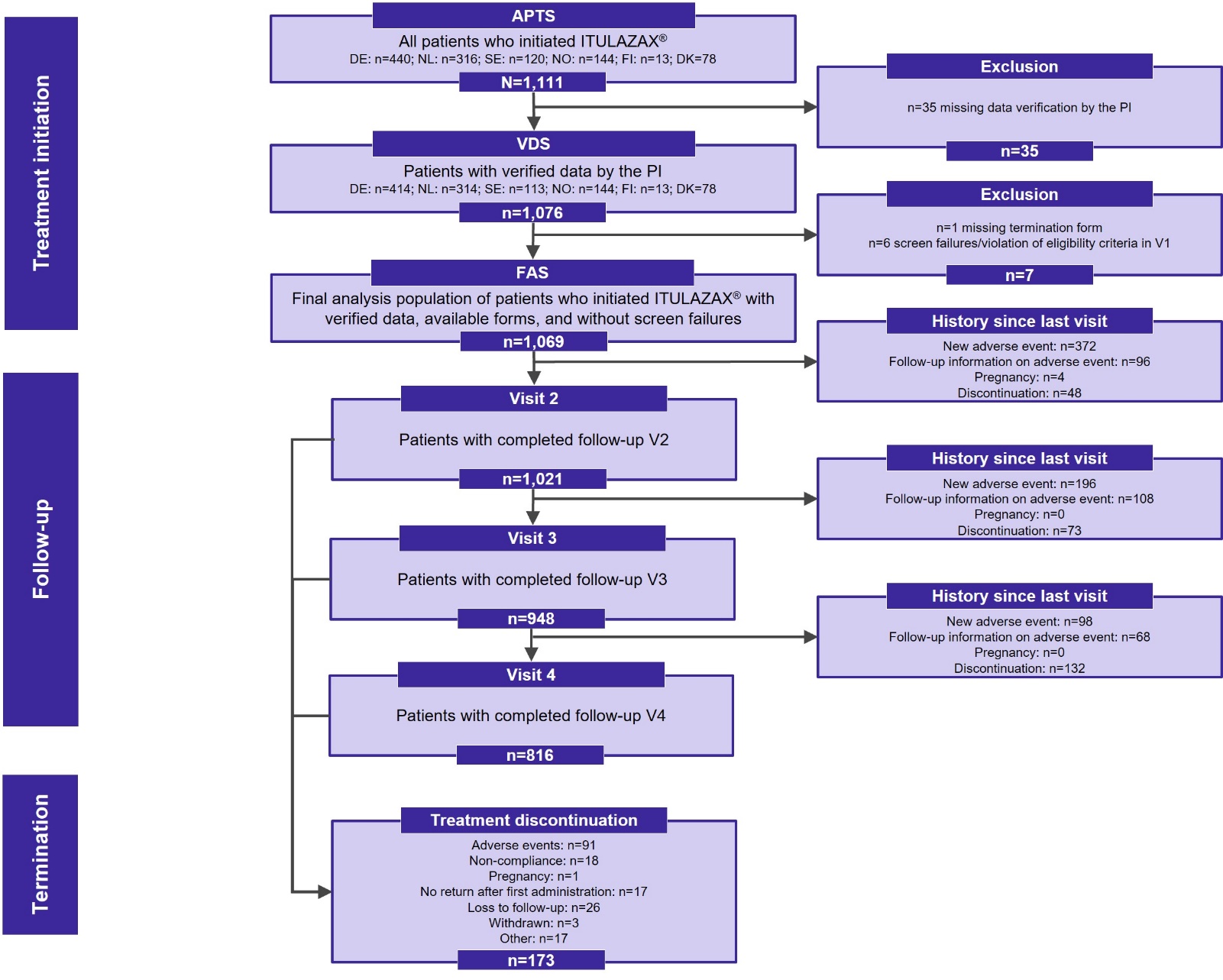


**Figure S2. A**: Percentage of patients with ADRs (at least one ADR with total incidence ≥1%) classified by severity during the observation period of the study for week 1-30 of treatment. **B**: data of A with maximum percentage of patients of 3.5% on y-axis for week 2-30.

**
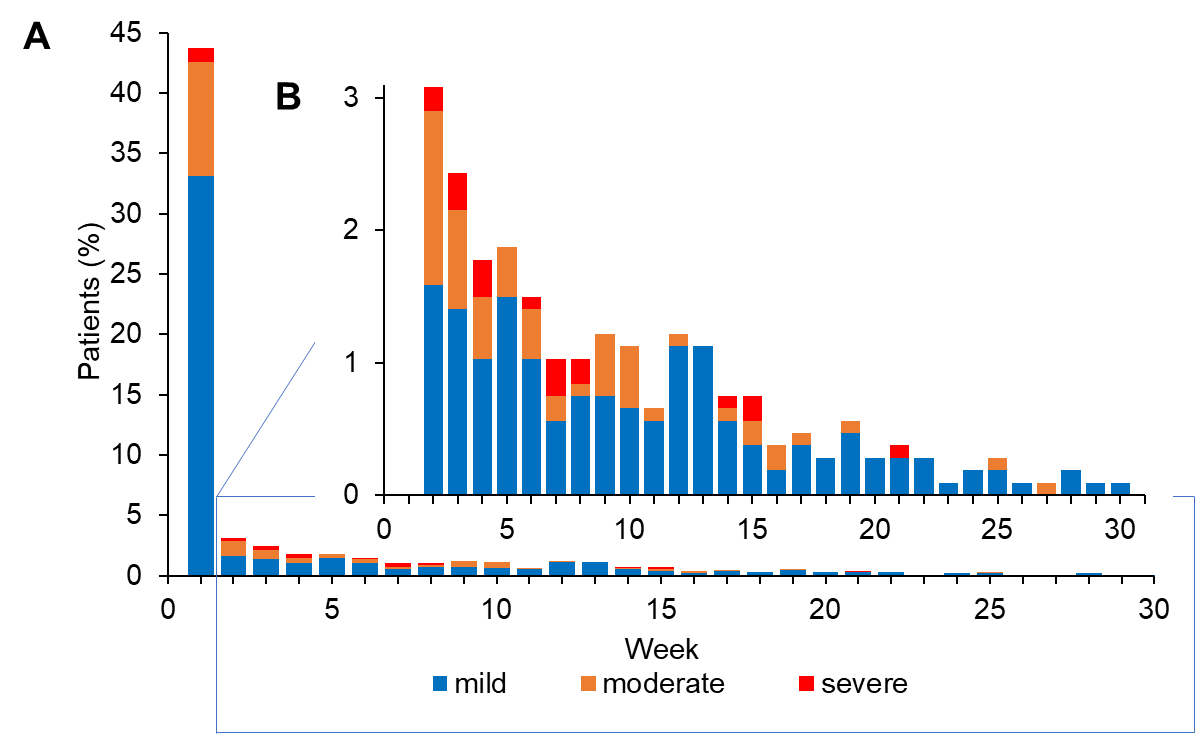
**

**Figure S3.** Change in symptoms (n=702 patients) at the individual last visit within the estimated tree pollen seasons vs. baseline. Periods of the tree pollen seasons were estimated for Germany, Denmark, and The Netherlands as: February to June 2021, January to June 2022, and Finland, Norway, and Sweden as: March to July 2021, February to July 2022.
